# Role of Protein Phosphorylation in the Regulation of Cell Cycle and DNA-Related Processes in Bacteria

**DOI:** 10.3389/fmicb.2016.00184

**Published:** 2016-02-16

**Authors:** Transito Garcia-Garcia, Sandrine Poncet, Abderahmane Derouiche, Lei Shi, Ivan Mijakovic, Marie-Françoise Noirot-Gros

**Affiliations:** ^1^Micalis Institute, INRA, AgroParisTech, Université Paris-SaclayJouy-en-Josas, France; ^2^Systems and Synthetic Biology, Department of Chemical and Biological Engineering, Chalmers University of TechnologyGothenburg, Sweden; ^3^Novo Nordisk Foundation Center for Biosustainability, Technical University of DenmarkHørsholm, Denmark

**Keywords:** protein phosphorylation, signaling, DNA replication, bacterial cell cycle, bacteria cell division

## Abstract

In all living organisms, the phosphorylation of proteins modulates various aspects of their functionalities. In eukaryotes, protein phosphorylation plays a key role in cell signaling, gene expression, and differentiation. Protein phosphorylation is also involved in the global control of DNA replication during the cell cycle, as well as in the mechanisms that cope with stress-induced replication blocks. Similar to eukaryotes, bacteria use Hanks-type kinases and phosphatases for signal transduction, and protein phosphorylation is involved in numerous cellular processes. However, it remains unclear whether protein phosphorylation in bacteria can also regulate the activity of proteins involved in DNA-mediated processes such as DNA replication or repair. Accumulating evidence supported by functional and biochemical studies suggests that phospho-regulatory mechanisms also take place during the bacterial cell cycle. Recent phosphoproteomics and interactomics studies identified numerous phosphoproteins involved in various aspect of DNA metabolism strongly supporting the existence of such level of regulation in bacteria. Similar to eukaryotes, bacterial scaffolding-like proteins emerged as platforms for kinase activation and signaling. This review reports the current knowledge on the phosphorylation of proteins involved in the maintenance of genome integrity and the regulation of cell cycle in bacteria that reveals surprising similarities to eukaryotes.

## Introduction

In all living cells, many cellular processes are controlled through the reversible phosphorylation of proteins on serine, threonine and tyrosine (Ser/Thr/Tyr) which results from the opposing action of kinases and phosphatases. While the role of Ser/Thr and Tyr -protein kinases (STYKs) and phosphatases in cellular regulations is extensively documented in eukaryotes, the Ser/Thr/Tyr signaling network in bacteria has a more recent history (Hunter, 2000; Pereira et al., 2011; Dworkin, 2015; Kalantari et al., 2015; Manuse et al., 2015). Analysis of microbial genomes revealed that Hanks-type Ser/Thr kinases (eSTKs) and phosphatases (eSTPs) are also widespread in bacteria (Zhang et al., 2007; Zhu et al., 2014). However, prokaryotic Tyr phosphorylation is carried out by a different family of enzymes, the bacterial tyrosine kinases or BY-kinases, which do not share the structural homology with eukaryotic STYKs. Instead, BY-kinases possess structural features characteristic of P-loop ATPases. BY-kinases as well as eSTKs have been shown to regulate cell-cycle events, cell adaptation to environmental cues, and virulence in many species (Wright and Ulijasz, 2014; Zhu et al., 2014; Kalantari et al., 2015).

Phosphorylation provides a sensitive and dynamic way to regulate protein activity, stability, protein interaction and sub-cellular localization. Global regulators are often targeted by more than one kinase, altering the expression of many genes. The phosphorylation of the transition state transcription regulator AbrB of *Bacillus subtilis* by three Ser/Thr kinases, PrkC, PrkD, and YabT, leads to the deregulation of numerous target genes during the transition of vegetative to stationary growth (Kobir et al., 2014). In the human pathogen bacteria *Staphylococcus aureus*, the phosphorylation of the global regulator MgrA also modulates gene expression in a growth phase-dependent manner (Truong-Bolduc et al., 2008). By adding a local negative charge, phosphorylation can positively or negatively affect the DNA binding of transcriptional factors to their regulatory sequences, thus participating in eliciting the cellular response. A recent example of tyrosine phosphorylation-mediated positive regulation is illustrated by the regulator SalA, which undergoes a conformational change upon phosphorylation at tyrosine 327 that enhances its DNA binding affinity for *scoC* upstream regulatory sequences (Derouiche et al., 2015a). Conversely, phosphorylation of the virulence regulator SarA of *S. aureus* at serine 75 within the HTH-DNA binding region negatively modulates its ability to bind DNA leading to the negative regulation of gene expression (Didier et al., 2010).

It was also observed that modulation by phosphorylation of the DNA binding of transcriptional factors plays a role in the regulation of chromosomal replication. The two component regulators MtrA of *Mycobacterium tuberculosis* and CtrA of *Caulobacter Crescentus* are illustrative examples that phosphorylation acts to temporally restrain access of the replication initiator protein DnaA to the origin DNA sequence *oriC* (Didier et al., 2010; Pini et al., 2015). A similar strategy was proposed to account for the role of the master regulator Spo0A in negatively controlling DNA replication initiation by inhibiting the DnaA-dependent DNA duplex unwinding during sporulation in *Bacillus subtilis* (Castilla-Llorente et al., 2006; Xenopoulos and Piggot, 2011; Boonstra et al., 2013). Hence, taking advantage of the versatility of transcription factors combined with the limited time frame during which they contact their regulatory sequence, bacterial cells have evolved multifunctional regulatory proteins able to act in both gene expression and replication initiation control.

However, little is known about the role of phosphorylation in directly regulating the DNA-binding activity of proteins involved in other DNA-mediated processes. Large-scale phosphoproteome studies have been conducted in bacteria, covering gram-negative as well as gram positive species (Macek et al., 2007, 2008; Voisin et al., 2007; Soufi et al., 2008; Ravichandran et al., 2009; Prisic et al., 2010; Schmidl et al., 2010; Ge et al., 2011; Manteca et al., 2011; Misra et al., 2011; Bai and Ji, 2012; Elsholz et al., 2012; Esser et al., 2012; Hansen et al., 2013; Soares et al., 2013; Yang et al., 2013; Kennelly, 2014; Ortega et al., 2014; Ravikumar et al., 2014; Nakedi et al., 2015; Pan et al., 2015). These databases reveal an increasing number of bacterial proteins phosphorylated on Ser/Thr/Tyr, involved in a variety of cellular processes related to DNA metabolism (Pan et al., 2015). Recently, a protein-protein interaction (PPI) network centered on *B. subtilis* S/T/Y protein kinases and phosphatases emerged as a powerful tool in bacterial signal transduction research, highlighting the existence of many regulatory pathways controlled by phosphorylation during DNA replication, chromosomal segregation and cytokinesis (Shi et al., 2014b). As with transcription factors, we can anticipate that phosphorylation could modulate protein-binding properties to DNA as well as to other proteins. Therefore, a great challenge is now to understand the role of protein phosphorylation in the coordination and integration of the different DNA processes during the bacterial cell cycle. In this review we will focus on novel aspects of regulation in bacteria that resemble those taking place in eukaryotes. We will highlight the growing evidence for phosphorylation of proteins involved in many DNA-related processes and the existence of scaffold proteins that act as signaling integrators by facilitating interaction and co-localization of kinases and their targets.

## Identification of phospho-proteins involved in DNA-related processes in bacteria

In living cells, the genome is continuously exposed to endogenous and exogenous damaging agents. If not repaired, the replication of damaged chromosomes can cause fork stalling or collapse and subsequent genome instability and possibly cell death (De Septenville et al., 2012; Merrikh et al., 2012; Cortez, 2015; Dungrawala et al., 2015). Sensing and repairing DNA damage is therefore necessary to ensure chromosome integrity. Upon detection of damage, eukaryotic cells relay information through signal transduction cascades to coordinate a biological response including cell cycle arrests and DNA repair pathways (Iyer and Rhind, 2013). These signaling cascades generally involve the successive activation of protein kinases, punctuated by the phosphorylation of protein effectors of DNA damage checkpoint and processing (Subramanian and Hochwagen, 2014). These checkpoints involve sensor proteins that identify DNA-related disorders, converting them into phosphorylation events that modulate the functions of specific target proteins. In eukaryotes, the evolutionary conserved ATM (ataxia-telangiectasia mutated) and ATR (ATM and Rad 3-related) Ser/Thr kinases are considered a paradigm for such transduction cascades (Cimprich and Cortez, 2008). These two master regulators act in response to double strand breaks (ATM), as well as in replication initiation control and repair of damaged forks (ATR) to coordinate cell cycle, repair, and replication (Shechter et al., 2004a,b; Cimprich and Cortez, 2008; Lee et al., 2015).

In most bacteria, the replication of the circular chromosome requires the coordinated action of conserved proteins to perform the initiation, elongation and termination steps (Langston and O'Donnell, 2006; Langston et al., 2009). Initiation is mediated by the replication initiator protein DnaA, which binds to specific origin sequences. This forms a nucleoprotein-structure leading to the unwinding of the duplex DNA for subsequent loading of the replication machinery or replisome. A bacterial replisome is usually composed of a type III DNA polymerase, a DNA helicase, and a sliding clamp with an associated clamp loading protein complex. During elongation, the replication factory encounters numerous road blocks or damage that can cause fork arrests (Pomerantz and O'Donnell, 2010; Mettrick and Grainge, 2015). As in eukaryotes, mechanisms are required to repair and/or restart the ongoing replication fork to preserve genomic integrity (Merrikh et al., 2012). In all living organisms, chromosome replication must be coordinated with other cellular pathways (Sclafani and Holzen, 2007).

**Figure 1:**
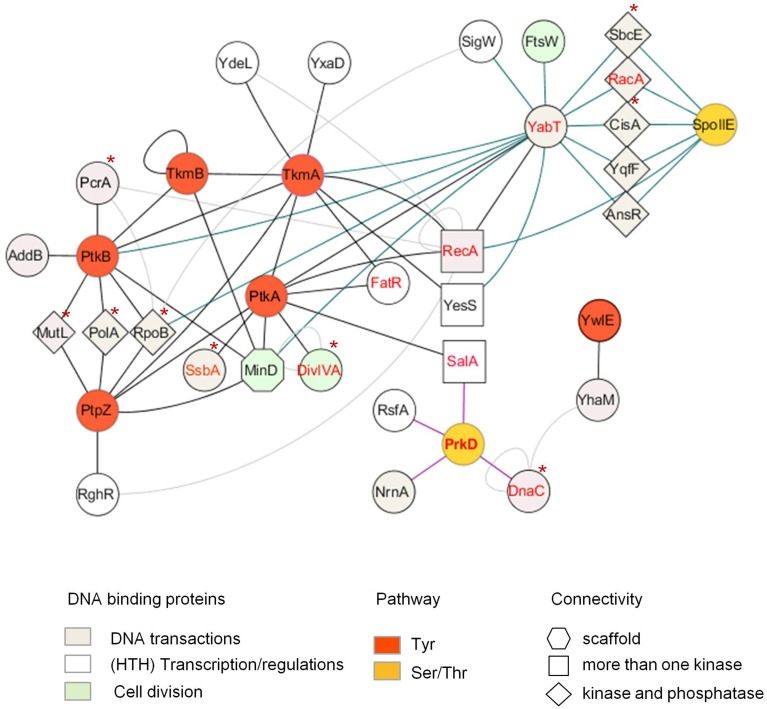
**PPI network centered on ser/thr (yellow nodes, bleu edges) and tyr (red nodes, black edges) kinases, phosphatases and modulators of *B. subtilis* reveals potential regulations by phosphorylation of various DNA-related pathways**. Proteins are represented as nodes connected by edges. Potential substrates, defined as connected by both a kinase and cognate phosphatase, are represented by diamonds. HTH-containing proteins, connected by more than one kinase, are represented by squares; *In vitro* characterized phospho-proteins are labeled in red. Proteins found phosphorylated in other bacteria are indicated by an asterix. Other interactions between the proteins are illustrated by light gray edges (from Marchadier et al., 2011; Shi et al., 2014b). *In vitro* validated kinase-substrate phosphorylation is indicated by dashed lines.

In bacteria, however, whether phosphorylation events play a role in sensing replication stresses remains to be ascertained. Recent studies highlight kinase activation by cross-phosphorylation in *B. subtilis* and *M. tuberculosis*, suggesting the existence of bacterial signaling cascades comparable to those found in eukaryotes (Baer et al., 2014; Shi et al., 2014a). Although the complement of proteins targeted by these potential regulatory cascades are not yet known, increasing numbers of large scale mass spectrometry-based proteomics studies find that proteins involved in various DNA-related processes are also phosphorylated in bacteria (Macek et al., 2007, 2008; Voisin et al., 2007; Soufi et al., 2008; Ravichandran et al., 2009; Prisic et al., 2010; Schmidl et al., 2010; Ge et al., 2011; Manteca et al., 2011; Misra et al., 2011; Bai and Ji, 2012; Elsholz et al., 2012; Esser et al., 2012; Hansen et al., 2013; Soares et al., 2013; Yang et al., 2013; Kennelly, 2014; Ortega et al., 2014; Ravikumar et al., 2014; Nakedi et al., 2015; Pan et al., 2015; Table 1). Another approach, based on yeast two-hybrid detection of protein binding partners of *B. subtilis* protein kinase and phosphatase, identifies numerous potential DNA-binding protein substrates, some of them already characterized as phosphoproteins in other studies, or further validated *in vivo* and/or *in vitro* (Shi et al., 2014b; Table 2, Figure 1). This study lays the foundation for investigating the role of phosphorylation in modulating DNA-related processes in this bacteria.

**Table 1:** **Phosphorylation of proteins involved in DNA-dependent machineries and processes in bacteria**.

**Pathway**	**Target**	**KO**	**Function**	**Tyr-P**	**Ser/Thr-P**
DNA repair and recombination	MUTL	K03572	DNA Mismatch repair factor	*MUTL_BACSU; B1XQB2_SYNP2*	*B1XQB2_SYNP2*
	MUTS	K03555	DNA Mismatch repair protein	*B1XM45_SYNP2*
	MUTM	K10563	FPG/DNA-N glycosylase	*B1XQB2_SYNP2*	*B1XQB2_SYNP2*
	UVRB	K03702	UvrABC system protein B	*UVRB_STRP2*
	UVRD	K03657	ATP-dep. DNA helicase (PcrA)	*PCRA_BACSU; O26013_HELPY*	*O26013_HELPY; Q3K0V8_STRA1*
	RUVX	K07447	Holliday junction resolvase	*RUVX_ECO57*
	RECA	K03553	SOS repair/DNA processing	*RECA_BACSU; RECA_ECO57*	*RECA_BACSU; RECA_THET8*
	RECG	K03655	ATP-dependent DNA helicase	*RECG_MYCTO*
	MFD	K03723	Transcription-repair factor	*B1XL29_SYNP2*	*Q04N58_STRP2; B1XL29_SYNP2*
	RECQ	K03654	ATP-dependent DNA helicase	*B1XQJ2_SYNP2*
	REP	K03656	ATP dependent DNA helicase	*Q3K4P8_PSEPF*
	DINI	K1214*9*	DNA-damage-inducible	*DINI_ECO57*
	RECT	K07455	DNA-Binding protein	*RECT_ECOLI*
	EX1	K01141	Exodeoxyribonuclease I (sbsB)	*Q8X8T9_ECO57; Q9HW85_PSEAE*	*Q9HW85_PSEAE*
	EX3	K01142	Exodeoxyribonuclease III	*EX3_ECOLI*
	EX7L	K03601	Exodeoxyribonuclease VII (A)	*EX7L_THETN*
	EX7S	K03602	Exodeoxyribonuclease VII (B)	*EX7S_STRA1*
	RADA	K04485	DNA repair protein (radA, sms)	*Q8XB28_ECO57*, *B1XJC1_SYNP2*	*B1XJC1_SYNP2*
	SSBB	Single-stand binding protein B	*SSBB_BACSU*
DNA replication	SSBA	K03111	Single-stand binding protein A	*SSBA_BACSU;*	Q04JK9_STRP2*; SSBA_BACSU*
	DPO1	K02335	DNA polymerase I	*DPO1_BACSU; B1XL22_SYNP2*	*B1XL22_SYNP2; DPO1_ECOLI*
	LIGA	K01972	DNA ligase A	*DNLJ_LISMO; DNLJ_ECOLI*
	DNAA	K02313	DNA replication initiator	*DNAA_STRCO*
	DPO3	K02337	Replicative DNA polymerase PolC	*HOLE_ECO57*
	DNAB	K02314	Replicative DNA helicase	*B1XQ85_SYNP2*	*B1XQ85_SYNP2; DNAC_BACSU*
	DPO3A	K02337	DNA polymerase III (θ su)	*HOLE_ECO57*
	TUS	K10748	DNA replication terminus protein	*TUS_ECO57*
Nucleoid structure and DNA condensation	GYRB	K02470	DNA gyrase β subunit	*GYRB_ECO57*	*GYRB_BACSU*
	TOP1	K03168	DNA topoisomerase 1	*TOP1_MYCTO*; *Q8X7C5_ECO57*	*TOP1_MYCPN*
	PARC	K02621	DNA topoisomerase 4 suA	*PARC_PSEAE*
	NAP	K09747	Nucleoid associated protein	*B1XLF8_SYNP2*;
	DBH1	K03530	DNA-binding protein HupA	*DBH1_BACSU; Q92A74_LISMO; DBH1_STRCO; B1XQQ7_SYNP2*
	DPS	K04047	DNA protection during starvation	*DPS_ECOLI; DPS_ECO57*	*DPS_ECOLI; DPS_CAMJE; DPS_LISMO; Q3K7V3_PSEPF*
	DBHB	K03530	DNA-binding protein HupB	*DBHB_ECOLI; Q6N5M1_RHOPA*
	HNS	K03746	DNA-binding protein H-NS	*HNS_ECOLI; HNS_ECO57*	*HNS_ECOLI*
	STPA	K11685	DNA-binding protein StpA	*CNU_ECO57*, STPA_ECOLI
	SMC	K03529	Chromosome partition protein	Q84EC7_SYNP2*; SMC_MYCTO*
Chrom. segregation	PARA	K03496	ATPase, ParA-family	B1XQR5_SYNP2
	FTSK	K03466	DNA translocase FtsK	*FTSK_ECO57; FTSK_ECOLI*	*FTSK_MYCTO*
Restrictionmodification	B1XQZ6	K01156	Type III R/M-Helicase	*B1XQZ6_SYNP2*
	ECO57IR	K00571	Type IIS R/M-methyltransferase	*O26046_HELPY*	*Q04K98_STRP2*
	HSDM	K03427	Type I restriction enzyme M	*O25953_HELPY*	O33298_MYCTU
DNA transcription	RPOB	K03043	RNA polymerase β subunit	*RPOB_BACSU; RPOB_ECO57*	*RPOB_ECOLI; RPOB_MYCTORPOB_CLOAB; RPOB_STRP2; RPOB_BACSU*
	RPOC	K03046	RNA polymerase β' subunit	*RPOC_BACSU; RPOC_ECO57*	*RPOC2_SYNP2; RPOC_CLOAB; RPOC_BACSU;* C4WZY6_KLEPN *RPOC_CLOAB; RPOC_STRP2*
	RPOBC	K13797	*RPOBC_HELPY;*	*RPOBC_HELPY*
	RPOA	K03040	RNA polymerase α subunit	*RPOA_ECO57; RPOA_ECOLI; C4X0R2_KLEPN*	*RPOA_STRP2; RPOA_CLOAB*
	RPOE	K03088	RNA polymerase σ factor H	*RPOE_MYCPN; SIGH_MYCBO; SIGH_MYCTO*
Other		K03722	DnaQ exonuclease/DinG helicase		Q04L99_STRP2
			Putative helicase		*B1XR86_SYNP2*
		K03578	ATP dependent helicase HrpA	Q3KGE3_PSEPF	
		K04763	Putative DNA integrase/recombinase		*B1XQW1_SYNP2*

*Escherichia coli K12 and O157:H7 species (ECOLI and ECO57:H7) (Macek et al., 2008; Hansen et al., 2013; Soares et al., 2013), Helicobacter pylori (HELPY) (Ge et al., 2011), Campylocacter jejuni (CAMJE) (Voisin et al., 2007), Bacillus subtilis (BACSU) (Macek et al., 2007; Jers et al., 2010; Elsholz et al., 2012; Shi et al., 2014b), Clostridium acetobutilicum (CLOAB) (Bai and Ji, 2012), Listeria Monocytogenes (LISMO) (Misra et al., 2011), Pseudomonas fluorescens Pf0-1 (PSEPF) (Ravichandran et al., 2009), Streptococcus agalactiae serotype 1a (STRA1) (Burnside et al., 2011), Streptococcus pneumoniae serotype 2 (STREP2) (Sun et al., 2010), Streptococcus coelicolor (STREPCO) (Manteca et al., 2011), Thermus thermophilus HB8 (THET8) (Takahata et al., 2012), Thermoanaerobacter tengcongensis (THETN) (Lin et al., 2012), Mycobacterium tuberculosis (MYCTO and MYCTU) (Prisic et al., 2010), Mycobacterioum bovis (MYCBO) (Nakedi et al., 2015), Mycoplasma pneumoniae (MYCPN) (Schmidl et al., 2010), Synechococcus sp. (SYNP2) (Yang et al., 2013). http://dbpsp.biocuckoo.org/browse.php (Pan et al., 2015). Protein target names are given according to HAMAP standard (http://hamap.expasy.org) and UniProtKB (http://www.uniprot.org/uniprot). Phosphoproteins provided with links to the dbPSP database (http://dbpsp.biocuckoo.org/) are labeled in blue. Phosphoproteins identified by other approaches are indicated in black. KO identifiers are provided for each family of proteins with links to the Kegg database for functional information (Kanehisa et al., 2012)*.

**Table 2:** **DNA-binding proteins targeted for phosphorylation in *B. subtilis***.

**Acc**	**Name**	**Function**	**PK**	**PP**	**P-sites**	**Val**	**Orth**	**References**
**TYROSINE PHOSPHORYLATION**								
P23477	ADDB	ATP-dependent deoxyribonuclease (B)	PtkB			–		1
P49850	MUTL	DNA mismatch repair factor	PtkB	PtpZ		a,d	Y	1
O34996	DPO1	DNA polymerase I	PtkB	PtpZ		a,d	T	1
O34580	UVRD	ATP-dependent DNA helicase (PcrA)	PtkB			d	Y,S,T	1
P16971	RECA	SOS repair factor/DNA processing	PtkA			b^#^,d	Y	1,2,3
P37870	RPOB	RNA polymerase β subunit	PtkB	PtpZ	Y_695_	a,c,d	Y,S,T	1,4
P37871	RPOC	RNA polymerase β' subunit			Y_338_	D		4
P37455	SSBA	Single-Stand Binding protein A	PtkA		Y_82_	b^*#^,c,d	S	5,6
O31903	YORK	Putative SPBc2 ss-DNA exonuclease	PtkA		Y_3∕11∕168∕220∕368∕473_	d		6,7
**SERINE/THREONINE PHOSPHORYLATION**								
P17867	CISA	Site-specific DNA recombinase	YabT	SpoIIE		a		1
P16971	RECA	SOS repair factor/DNA processing	YabT	SpoIIE	S_2_	a,b,c,d	Y	1,2,3
P45870	RACA	Chromosome-anchoring protein	YabT	SpoIIE		a, b^#^		1
O08455	SBCE	DSB repair	YabT	SpoIIE		a		1
P46344	YQFF	Putative Phosphodiesterase	YabT	SpoIIE		a		1
P37469	DNAC	Replicative DNA helicase DnaB-like	PrkD			b^#^,d	Y	1
P08821	DBH1	DNA-binding protein HU-1			T_4_, T_65_, S_74_	c,d	S,T	6
P05652	GYRB	DNA gyrase subunit B			S_400_	c,d	Y	2
P94590	SSBB	Single-stand DNA binding protein B	PtkA		Y_82_,T_52_	c,d	S	4
P37871	RPOB	RNA polymerase β subunit			S_314_	c,d	S,T,Y	4
P37871	RPOC	RNA polymerase β' subunit			S_339_	c		4

Validation (Val) is provided by (a) ability to interact with both a protein kinase (PK) and a cognate protein phosphatase (PP), (b) phosphorylation confirmed in vivo (*) and/or in vitro (^#^), (c) identified phosphosite(s), and (d) existence of phosphorylation at tyrosine (Y) serine (S) or threonine (T) in other bacterial orthologs (orth). 1 (Shi et al., 2014b); 2 (Soufi et al., 2008); 3 (Bidnenko et al., 2013); 4 (Elsholz et al., 2012); 5 (Mijakovic et al., 2006); 6 (Macek et al., 2007); 7 (Jers et al., 2010).

An example of phosphorylated multifunctional protein playing a role in various DNA processes is the single-stranded DNA-binding protein SSB (Mijakovic et al., 2006; Vujaklija and Macek, 2012). In bacteria, SSB proteins assemble onto DNA as homodimers or tetramers, each unit composed of an N-terminal DNA binding domain and a C-terminal intrinsically disordered tail that mediates interaction with numerous proteins (Kozlov et al., 2015). The bacterial SSB proteins are mainly associated with the replication machinery at the replicating forks and serve as central hubs to coordinate DNA replication and repair (Lecointe et al., 2007; Costes et al., 2010; Antony et al., 2013; Bentchikou et al., 2015). As in bacteria, the eukaryotic single strand binding protein RPA has multiple roles in protecting ssDNA, sensing and promoting repair of DNA damage via PPIs (Oakley and Patrick, 2010; Maréchal and Zou, 2015). In particular, phosphorylation of RPA negatively regulates its binding to DNA as well as its interaction with protein partners (Binz et al., 2003; Oakley et al., 2003). *B. subtilis* possesses two single strand binding proteins SSBA and B, respectively phosphorylated at a tyrosine, identified as Tyr82 in SSBA (Mijakovic et al., 2006). Additionally, a phospho-site at Thr52 was also identified in SSBB (Table 1; Jers et al., 2010; Elsholz et al., 2012). Examination of the 3D- structure of the SSB/DNA complex shows that Tyr82 (as well as the Thr52 neighbor residue Trp54) are involved in stacking with DNA (Yadav et al., 2012). This strongly supports the observation that phosphorylation of SSBA at residue Tyr82 negatively modulates its binding onto DNA *in vitro* and regulates the cell response to DNA damage (Mijakovic et al., 2006). Therefore, the role of SSB protein phosphorylation in modulating the cellular DNA damage response might be evolutionarily conserved in eukaryotes and in prokaryotes.

Another strong case was made for the interaction of the DNA replicative helicase DnaC by the Ser/Thr kinase PrkD, validated by *in vitro* assays and high correlation of co-expressions (Shi et al., 2014b). Phosphorylation of numerous components of the DNA replication machinery, as well as of proteins involved in the different steps of the replication cycle, have been identified in one or more bacteria among *Escherichia, Pseudomonas, Helicobacter, Camplylobacter, Listeria, Bacillus, Mycoplasma, Clostridium, Deinoccocus, Synechoccus*, and *Streptococcus* genus (Tables 1, 2). Orthologs of the highly conserved DNA polymerase PolA, DNA helicase DnaC and single stranded binding protein SSB were found phosphorylated, potentially reflecting the existence of generic regulatory mechanisms to control their activity across species.

The conservation of phosphorylation within protein domain families is indicative of proteins with high regulatory potential (Maathuis, 2008). Remarkably, several phosphoproteomic analyses have reported phosphorylation of the two largest subunits β and β' of the RNA polymerase machinery at serine, threonine or tyrosine, in seven bacterial species (Table 1). In eukaryotes, the recruitment of factors to the elongating RNAPII is regulated by the differential and dynamic phosphorylation of serine, threonine and tyrosine residues within the C-terminal domain of the Rpb1 large subunit during the transcription cycle (Heidemann et al., 2013). Although the existence of phosphorylation of the main components of the RNA polymerase in both bacteria and eukaryotic cells does not imply similar regulatory mechanisms, it does suggest that evolutionary constraints on regulating gene expression led to targeting the transcription machinery for modulating its activity and its ability to interact with other factors.

## Biological role of phospho-proteins in coordinating DNA- metabolism with transition state and cellular development in *B. subtilis*

A large panel of proteins involved in various DNA replication and repair pathways are identified with phospho-sites, suggesting that phosphorylation could play a role in coordinating the different cellular responses to DNA damage and integrity of the replicating chromosome (Table 1). Regulation by phosphorylation of DNA-related processes has been documented in *B. subtilis*. One process involves the DNA recombinase RecA, a multifunctional protein playing important roles in homologous recombination and repair of DNA double-strand breaks, as well as initiating the cellular SOS response to DNA damage in bacteria (Kuzminov, 2001; Chen et al., 2008; Butala et al., 2009). Recently, RecA was described as playing a role in monitoring and maintaining chromosome integrity at the onset of spore development (Bidnenko et al., 2013). This novel activity is regulated by phosphorylation by the ssDNA-dependent Ser/Thr kinase YabT, expressed during sporulation. Although the molecular mechanism underlying this activity is not yet understood, RecA phosphorylation at the N-terminal S2 plays a role in temporarily restraining sporulation furtherance upon encountering DNA damages. From the recent finding that RecA is also subjected to tyrosine phosphorylation by PtkA, we can anticipate a more complex picture for the regulation of its activities in *B. subtilis* (Shi et al., 2014b).

The DNA-binding developmental protein RacA illustrates a prospective role of phosphorylation in coordinating chromosome segregation with the asymmetric division taking place during sporulation in *B. subtilis*. During sporulation, RacA anchors the segregating chromosome at the cell pole of the forespore in a DivIVA-dependent manner (Ben-Yehuda et al., 2003; Wu and Errington, 2003; Ben-Yehuda et al., 2005; Perry and Edwards, 2006). In the *B. subtilis* PPI network, RacA is connected by both the Ser/Thr kinase YabT and cognate phosphatase SpoIIE, suggesting it may be a substrate of both (Shi et al., 2014b). This assumption, further confirmed *in vitro*, was also supported by the existence of a high positive correlation of expression of *racA* and *spoIIE* genes across numerous physiological conditions, indicating they are part of the same biological processes.

Nucleoid associated proteins (NAPs) are histone-like proteins involved in modifying the structural organization of chromosomes by changing its topology at a global or local level (Dillon and Dorman, 2010). As a consequence of their DNA-binding properties, NAP proteins are found to modulate different DNA-processes and act as global regulators of gene expression (Dorman, 2013). NAP proteins are diverse and present in all living cells (Dillon and Dorman, 2010; Dorman, 2013). The examination of phosphoproteomics data in the database of Phosphorylation Sites in Prokaryotes dbPSP reveals that many representatives of this class of proteins are phosphorylated, including HupA, HupB, H-NS (and H-NS like StpA), Dps and structural maintenance chromosome (SMC) proteins (Table 1). A particular feature of this class of proteins is the occurrence of phospho-sites across a broad spectrum of bacterial orthologs (Table 1), suggesting that they could be functionally regulated by phosphorylation. The recent discovery that phosphorylation of the histone-like protein HupB by STKPs kinases negatively modulates its ability to bind DNA in *M. tuberculosis* supports this assumption (Gupta et al., 2014). The phosphorylation state of the HupB protein correlates with the expression of *pknE* and *pknF* kinases. Their expression is high during the exponential growth phase and low when reaching the stationary phase. This differential phosphorylation strongly points to a potential mode of regulation of *HupB* which favors interaction with DNA during the stationary phase to promote compaction of the chromosome.

## MinD, a signaling scaffold-like protein

Kinases are regulated by forming specific associations with arrays of different proteins, including kinase inhibitors and activators, as well as scaffold and anchoring proteins that target the kinase-complexes to specific subcellular sites. Scaffold proteins are versatile hubs that spatially and temporally tether signaling components in eukaryotic cells. Interactome-based research in yeast provides a definition of classical signaling scaffold proteins as (i) having an active role in signaling while being itself devoid of catalytic activity relevant for signaling, and (ii) being able to promote the interaction of multiple components of signaling cascades at a particular cellular location to regulate their activity (Zeke et al., 2009). A eukaryotic scaffold protein paradigm is the Ste5 protein of the mitogen-activated protein kinase (MAPK) pathway in yeast, which insulates three protein kinases MAPKKK Ste11, MAPKK Ste7, and MAPK Fus3 to promote a signaling cascade (Good et al., 2009).

Recent report of a yeast two-hybrid based PPI network centered on tyrosine-kinase in *B. subtilis* provide a 2D map of potential BY-kinase interacting partners (Shi et al., 2014b). Numerous protein partners are contacted by cognate kinase/phosphatase pairs, supporting the hypothesis that they are substrates. However, there are exception to this rule. The bacterial cell division regulator MinD is contacted by both the tyrosine kinase PtkA and tyrosine phosphatase PtpZ in the PPI network, but not targeted for phosphorylation by PtkA (Shi et al., 2014b). Strikingly, PtkA has been found to phosphorylate the septum site selection protein DivIVA, which recruits MinD at the cell poles via MinJ. Subcellular localization studies reveal that PtkA localizes at the cell pole in a MinD-dependent fashion. Septum defect phenotypes are observed in absence of PtkA and aggravated in a *zapA* deficient background, strongly suggesting that PtkA could play a role during cell division. All these observations support the hypothesis that *B. subtilis* MinD, in addition to its characterized role in recruiting the FtsZ-negative regulator MinC to prevent the formation of a Z-ring at the cell poles (Gregory et al., 2008), also acts as an intracellular organizer of signaling components involved in the regulation of cytokinesis (Shi et al., 2014b).

**Figure 2:**
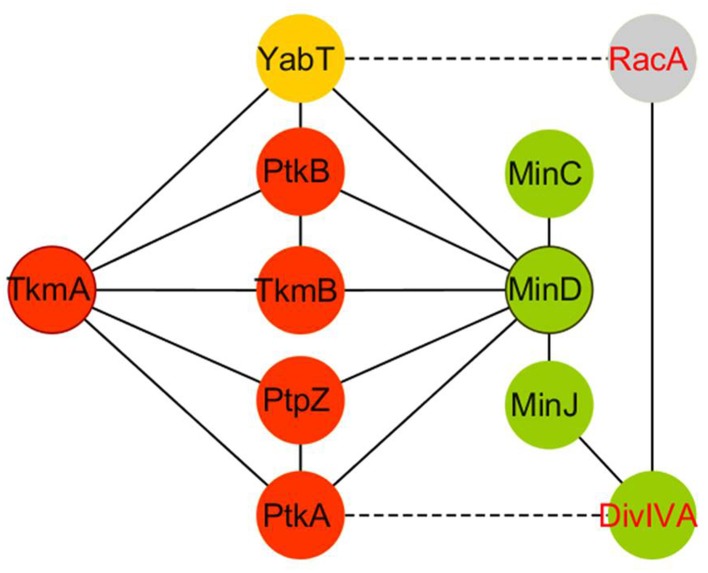
**PPI profile similarities between the protein modulators TkmA (anchor) and MinD (scaffold) in *B. subtilis***. Tyr and Ser/Thr phosphorylation pathways are filled in red and yellow, respectively. Cell division pathway is indicated in green.

MinD belongs to the class of ParA-like AAA+ ATPases and bears structural homology to PtkA (Derouiche et al., 2015b). However, it lacks the tyrosine cluster determinant for autophosphorylation and is thus not a catalytically active kinase. Importantly, similar to TkmA, the transmembrane and cognate modulator of PtkA, necessary for the general activation of the kinase in the cell, MinD binds to and activates PtkA. In the PPI network, MinD is also found to interact with the second *B. subtilis* BY-kinase PktB, the Ser/Thr Hanks kinase YabT and a phosphatase (PtpZ), suggesting a potential ability to bring various components of tyrosine, but also serine/threonine phosphorylation pathways in close proximity (Figure 2). In many ways, MinD fulfills the definition of a signaling protein scaffold that allows several actors belonging to different pathways to co-localize at the cell poles. Remarkably, in the *B. subtilis* PPI phosphotyrosine network, MinD exhibits an interacting landscape similar to that of TkmA (Shi et al., 2014b; Figure 2). As a transmembrane protein, TkmA is classified as a receptor protein, activating PtkA upon sensing external inputs, while MinD perfectly matches with the definition of a classical scaffold, enforcing the proximity of kinase/phosphatase and substrate at a specific time and location during the bacteria cell cycle. However, the potential ability of TkmA to contact more than one kinase, a phosphatase and various PtkA substrates suggests that this receptor could also act as a docking platform to activate tyrosine kinase signaling events (Jers et al., 2010).

MinD acting as a scaffold is therefore proposed to promote PtkA-mediated phosphorylation of DivIVA in *B. subtilis* (Shi et al., 2014b). Phosphorylation of DivIVA and other homologs by Ser/Thr kinases is described as playing a role in regulating apical growth and cell polarity in Actinobateria, as well as well as orchestrating peptidoglycan synthesis during cell elongation in streptococci (Fleurie et al., 2012; Hempel et al., 2012; Fleurie et al., 2014; Pompeo et al., 2015). A real question about the role of phosphorylation of DivIVA at tyrosine in regulating its dynamic from the cell poles to the future septum is now addressed in *B. subtilis*. In this bacterium, PtkA could modulate DivIVA at the cell pole through landing/activation by MinD. The potential presence of other signaling components, such as the phosphatase PtpZ, would also add to the fine-tuning of such regulations. The interaction of the sporulation YabT Ser/Thr kinase with MinD might also provide a clue toward understanding how DivVIA switches from a MinCD(J)-mediated control of FtsZ assembly during vegetative growth, to a RacA mediated chromosome anchorage to the pole of the fore spore compartment at the onset of sporulation (Ben-Yehuda et al., 2003; Wu and Errington, 2003; Ben-Yehuda et al., 2005; Perry and Edwards, 2006; Bramkamp et al., 2008). This novel function of MinD as a signaling scaffold is thus expected to participate in coordinating cytokinesis, the terminal step of the cell cycle, with other pathways during the different *Bacillus* life-styles.

MinD/ParA-like proteins are widely found distributed among bacteria. They are not only involved in different aspects of regulation of cell division and chromosome segregation, but also in polar localization of cellular machineries, such as those directing conjugative transfer or chemotaxis, as well as those organizing the polar positioning of Type IV pili (Kirkpatrick and Viollier, 2012; Lutkenhaus, 2012; Treuner-Lange and Søgaard-Andersen, 2014). ParA/MinD-like proteins are often described as protein-hubs responsible for the architectural and spatial organization of necessary components driving these processes (Lutkenhaus, 2012). In the asymmetrically dividing bacterium *C. crescentus*, the mechanisms underlying the segregation of the cell fate determinants to the two structurally distinct cell poles also requires MinD/ParA-like proteins to achieve polar localization of chromosomes and cell *division* (Thanbichler and Shapiro, 2006). The MinD-like ATPase MipZ forms a dynamic complex with the DNA partitioning protein ParB at the chromosomal origin of replication, and also negatively regulates the polymerization of FtsZ (Du and Lutkenhaus, 2012; Kiekebusch et al., 2012). Through ATP hydrolysis, MipZ acts as a molecular switch to couple chromosome segregation with the control of mid-cell positioning of the FtsZ ring in *C. crescentus* (Thanbichler and Shapiro, 2006). Although its role in signaling scaffold is not proven, recent studies highlight the importance of ParB phosphorylation in the localization and function of proteins involved in chromosome segregation in mycobacteria (Baronian et al., 2015). Lastly, the discovery that the ParA-like BY-kinase CspD, encoded by the cps capsular operon in *S. pneumoniae*, which plays a role in coordinating capsular polysaccharide with chromosome segregation through interaction with ParB, leads to the hypothesis that this kinase could act as molecular scaffold (Nourikyan et al., 2015). Although no proteins have so far been found to be phosphorylated by CpsD, ATP hydrolysis leading to CpsD autophosphorylation plays a major role in regulating the dynamic of localization of the ParB proteins bound to the chromosomal replication origin, and provides a molecular switch to couple the cell cycle with cell elongation (Nourikyan et al., 2015).

## Conclusions

Large-scale profiling of phosphoproteins and phospho-sites by mass spectrometry-based proteomics, differential phosphoproteomics across physiological conditions and kinase-related phosphoproteomes are helpful to identify kinase-specific substrates and understand the functional dynamics of phosphorylation networks. Additionally, interactomics identifies kinase-protein interactions revealing not only potential substrates, but also anchors and modulators. Hence, phosphoproteomics and interactomics are powerfull and complementary approaches to provide a blueprint of kinase and phosphatase-centered signaling networks. The ever growing data from large scale phosphoproteome studies in bacteria, coupled with identification of kinase, phosphatase, substrates, and modulators provides important insights into cellular regulations. It also provides a unique opportunity to understand how phosphorylation participates in the regulation of protein activities, their interactions with other proteins, and their localizations in the bacterial cell.

In many bacteria, phosphorylation of proteins involved in various DNA-related processes, together with biological evidence of their role during cell cycle, suggests the existence of checkpoint regulations akin to eukaryotes. In all living organisms, cell cycle events involve the temporal and spatial coordination of chromosome replication and segregation with cytokinesis, the entire process being also coordinated with cell growth. In eukaryotes, the existence of highly intricate signaling networks is ensured by quality control and checkpoint proteins that coordinate these sequential events and trigger cell cycle arrests when things go wrong. In bacteria, the different processes are more interwoven. Consequently, the accurate execution of each step relative to others in time and space is crucial and involves strict regulatory mechanisms. The finding, in *B. subtilis*, that cell division MinD may act as a signaling hub to reinforce the proximity of various components of the Tyr and Ser/Thr phosphorylation pathway, along with the septum site protein DivIA, again hints at the existence of scaffold-mediated signal transduction in bacteria. In addition, the recent discovery that bacterial kinase are themselves able to tune their activities through phosphorylation add to the complexity of the picture (Shi et al., 2014a). However, it also suggests that, just as in eukaryotes, such bacterial scaffolds could offer versatile strategies to dynamically coordinate cell processes via signaling.

## Author contributions

TG, SP, LS, and AD analyzed the bibliographic data. TG and SP provided the tables. MN wrote the manuscript. IM revised it critically. All authors gave final approval of the version.

## Funding

This work was supported by the grants from the Agence Nationale de la Recherche (2010-BLAN-1303-01) to MN and IM and the Vetenskapsrådet (2015-05319) and the Novo Nordisk Foundation to IM. TG is supported by the European Union, Marie Curie ITN AMBER, 317338.

### Conflict of interest statement

The authors declare that the research was conducted in the absence of any commercial or financial relationships that could be construed as a potential conflict of interest.
